# The action of β-hydroxybutyrate on the growth, metabolism and global histone H3 acetylation of spontaneous mouse mammary tumours: evidence of a β-hydroxybutyrate paradox

**DOI:** 10.1186/s40170-017-0166-z

**Published:** 2017-02-28

**Authors:** Loreta M. Rodrigues, Santiago Uribe-Lewis, Basetti Madhu, Davina J. Honess, Marion Stubbs, John R. Griffiths

**Affiliations:** Cancer Research UK Cambridge Institute, Li Ka Shing Centre, Robinson Way, Cambridge, CB2 ORE UK

**Keywords:** Ketone bodies, β-hydroxybutyrate, NEU/HER2 mammary tumours, Warburg effect, Histone acetylation, Magnetic Resonance Spectroscopy, Metabolites, Glycolysis, Oxidative phosphorylation

## Abstract

**Background:**

Ketone bodies have both metabolic and epigenetic roles in cancer. In several studies, they showed an anti-cancer effect via inhibition of histone deacetylases; however, other studies observed faster tumour growth. The related molecule butyrate also inhibits growth of some cancer cells and accelerates it in others. This “butyrate paradox” is thought to be due to butyrate mediating histone acetylation and thus inhibiting cell proliferation in cancers that preferentially utilise glucose (the Warburg effect); whereas in cells that oxidise butyrate as a fuel, it fails to reach inhibitory concentrations and can stimulate growth.

**Methods:**

We treated transgenic mice bearing spontaneous MMTV-NEU-NT mammary tumours with the ketone body β-hydroxybutyrate (β-OHB) and monitored tumour growth, metabolite concentrations and histone acetylation. In a cell line derived from these tumours, we also measured uptake of β-OHB and glucose, and lactate production, in the absence and presence of β-OHB.

**Results:**

β-OHB administration accelerated growth of MMTV-NEU-NT tumours, and their metabolic profile showed significant increases in ATP, glutamine, serine and choline-related metabolites. The β-OHB concentration within the treated tumours, 0.46 ± 0.05 μmol/g, had no effect on histone acetylation as shown by western blots. Cultured tumour cells incubated with 0.5 mM β-OHB showed β-OHB uptake that would be equivalent to 54% of glycolytic ATP phosphorylation and no significant change in glucose consumption or lactate production.

**Conclusions:**

These results suggest that a β-OHB paradox may occur in these mammary tumours in a manner analogous to the butyrate paradox. At low β-OHB concentrations (<1 mM, as observed in our tumour model post-treatment), and in the absence of a Warburg effect, β-OHB is consumed and thus acts as an oxidative energy source and not as an epigenetic factor. This would explain the increase in tumour growth after treatment, the metabolic profiles and the absence of an effect on histone H3 acetylation.

## Background

Over the last decade, there has been renewed interest in cancer metabolism, particularly with regard to the reprogramming of the metabolic networks within cancer tissue. Understanding these metabolic perturbations could open a window for therapeutic intervention. One such phenotype in cancer is the Warburg effect, often regarded as a characteristic feature of cancer cells and solid tumours [1] in which cancer cells perform aerobic glycolysis instead of the more metabolically efficient oxidative phosphorylation [2]. The consequent reliance of cancer cells on glucose has been exploited in metabolic therapies where calorie restriction and ketogenic diets have also been used to limit glucose metabolism and thus slow cancer progression [3].

The ketone bodies (KB), β-hydroxybutyrate (β-OHB) and acetoacetate are synthesised in the liver from acetyl-CoA and secreted into the blood, from which many tissues can take up and oxidatively metabolise them. Their main role is to provide an alternative substrate to glucose during prolonged starvation; in particular, due to their ability to cross the blood–brain barrier, they reduce the reliance of the brain on glucose. β-OHB has been proposed to be a unique nutritional compound since it has a calorific value more than glucose or pyruvate and more efficient provision of energy per molecule of oxygen compared to glucose, pyruvate and free fatty acids [4]. The metabolism of KB has effects on mitochondrial energetics and on brain metabolism that have therapeutic implications. Ketogenic diets have been used in refractory epilepsy and have provided some benefit in hypoxic states, for prevention of muscle-wasting and in cancer [4].

There have been several studies showing that administration of KB or the physiological state of ketosis had an anti-cancer effect [3, 5–7]. Paradoxically, however, in another group of studies KB were found to *promote* the growth of cancers [8–10]. On the anti-cancer side, studies on orthotopically implanted mouse astrocytomas have reported that malignant brain tumours are potentially manageable with dietary therapies that reduce glucose and elevate KB, since these brain tumours lack metabolic versatility and are dependent largely on glucose for energy [5]. More recent studies have evaluated the anti-cancer and anti-cachectic properties of KB in cultured pancreatic tumour cells as well as the effect of ketogenic diets on tumour burden and cachexia in orthotopically implanted models of pancreatic cancer [7]. The authors proposed that KB-induced metabolomic reprogramming by ketogenic diets suppresses cancer and cancer-induced cachexia. In another study, Poff et al. [3] showed that dietary administration of ketone precursors extended the survival time of mice with metastatic cancer by 50–70%. In contrast, other studies on breast cancer have shown that KB utilisation drives tumour growth and metastasis [8–10]. One of these papers demonstrated that KB, when administered systemically to animals bearing a breast tumour xenograft, promoted the growth of those tumours with no significant increase in angiogenesis and with a transcriptional shift towards oxidative mitochondrial metabolism in cancer epithelial cells relative to adjacent stromal cells [8].

In addition to their metabolic role, KB are also integrated into the regulation of epigenetic states and transcription, thus providing potential mechanisms that link cellular energy metabolism and regulation of gene expression via chromatin modification [11, 12]. β-OHB, for instance, is an endogenous inhibitor of the histone deacetylases (HDACs; also termed lysine deacetylases) which remove acetyl epigenetic marks from histones and other proteins that interact with DNA. Acetyl-CoA, in addition to being the substrate for synthesis of KBs, is also the co-factor of the histone acetyltransferase enzymes (HATs) that incorporate acetyl groups into histones [13]. Deregulation of histone acetylation results in abnormal expression profiles of genes involved in cell proliferation and differentiation and is associated with malignancy [14].

Butyrate, closely related to β-OHB, has been an essential agent for determining the role of histone acetylation in chromatin function, and observations that butyrate-treated cells show histone hyperacetylation led to the discovery that butyrate inhibits HDAC activity [15]. Its role in linking energy metabolism with epigenetics has been reported by Donohoe et al. [16]. Butyrate is a short-chain fatty acid produced by fermentation of dietary fibre in the colon, where it is metabolised oxidatively and functions as a primary energy source for colonocytes. Notably, butyrate has been shown to have growth-inhibitory effects in cancerous cells but either no effect or stimulation of growth in non-cancerous cells. These opposing effects on normal versus cancerous cells have been termed the butyrate paradox (reviewed by Lupton 2004) [17]. It is thought that this paradox results from epigenetic effects of butyrate on the two cell types. Donohoe et al. [16] suggest that the mechanism of butyrate-mediated histone acetylation and cell proliferation could be dictated by the Warburg effect. In cancer cells that exhibit the Warburg effect and therefore derive their energy from glycolysis, butyrate is metabolised inefficiently; it therefore accumulates and functions as an HDAC inhibitor. In normal cells, butyrate functions as an oxidative energy source, stimulating proliferation, and its concentration is thereby reduced below the level required for HDAC inhibition.

Not all cancer cells display the Warburg effect. For instance, HeLa cells can adapt their mitochondrial network structurally and functionally to derive energy exclusively by oxidation of glutamate [18] and in prostate cancer cells, fatty acid oxidation is a dominant bioenergetic pathway [19]. Furthermore, Lisanti has described a “reverse” Warburg effect in which cancer cells utilise lactate secreted by adjacent host cells [20]. It seems probable, therefore, that any cancer cells that normally utilise oxidative metabolism would be likely to oxidise butyrate in the same way as normal host cells and thus reduce their internal concentration of butyrate below the level required to induce HDAC inhibition so that they would not, therefore, display the butyrate paradox.

Previous studies on the action of KB on the growth of tumours have used transplanted tumours. These models have limitations, in particular for studies like the present one, with regard to the metabolic interactions between tumour cells and the adjacent “host” cells in the tumour microenvironment. The architecture of a transplanted tumour is different from that of a spontaneous tumour: subcutaneously implanted tumours, in particular, usually have poor blood supplies and therefore tend to be more hypoxic than spontaneous tumours, while many of the standard transplanted models have become selected, during repeated passages, to thrive in their unnatural environment. In addition, transplanted tumour models often utilise cells that have been pre-selected for rapid growth in an unnatural cell culture environment. These limitations are all circumvented by the use of genetically engineered models in which tumours arise spontaneously in the “autochthonous” tissue.

In the MMTV-NEU-NT transgenic mice used in the present study, tumours arise spontaneously in the mammary tissue by overexpression of the activated form of the NEU/HER2 oncogene, similar to the ERBB2 gene that is amplified in many human breast cancers [21, 22]. We have investigated the effect of β-OHB on the growth, energy metabolism and histone H3 acetylation of MMTV-NEU-NT spontaneous mammary tumours. We also investigated the effect of β-OHB on the uptake of glucose and lactate production in cultured cells of the same tumour type to get a measure of their glycolytic rate.

We have used both ^1^H and ^31^P magnetic resonance spectroscopy (MRS) to monitor metabolites in the tumours and livers of MMTV-NEU-NT transgenic mice that have been treated with β-OHB intraperitoneally (ip). MRS allows the simultaneous detection of numerous metabolites related to glucose, protein and lipid metabolism and is a valuable tool both in vivo and in vitro for detecting metabolic features of tumours and comparing them with those of normal tissue both during normal progression and after treatment [23]. Biomarkers for tissue bioenergetics, such as nucleoside triphosphate (NTP), inorganic phosphate (Pi), creatine (Cr) and phosphocreatine (PCr); glycolytic markers such as glucose and lactate; amino acids such as glycine, alanine, leucine, isoleucine, glutamine and glutamate; ketone bodies and intermediary metabolites like β-OHB, acetate, succinate and choline-containing phospholipid metabolites are all readily observed with MRS methods.

Activated choline metabolism is a hallmark of many cancers and is characterised by increases in the phosphocholine (PC) and total choline (tCho) peaks. That change, referred to as the choline phenotype, was discovered mainly in MRS studies on tumours [24]. Subsequent studies have identified malignant transformation rather than just cell proliferation as the cause of abnormal choline metabolism in cancers [25]. Choline-containing metabolites provide information on tumour membrane metabolism, since the phosphomonoesters (PMEs) PC and phosphoethanolamine (PE) are precursors of the phosphatidylcholine and phosphatidylethanolamine in biological membranes and the phosphodiesters (PDEs) glycerophosphocholine (GPC) and glycerophosphoethanolamine (GPE), are breakdown products of phosphatidylcholine and phosphatidylethanolamine.

## Methods

### Materials

DL-β-hydroxybutyric acid (sodium salt) was purchased from Sigma-Aldrich and made up in phosphate buffered saline (PBS) at 50 mg/ml, pH to 7.4 with 0.1 M hydrochloric acid. Perchloric acid (PCA) and potassium hydroxide were purchased from Merck (Poole, UK). Sodium 3-trimethylsilyl-2,2,3,3-tetra-deuteropropionate (TSP) was purchased from Goss Scientific Instruments Ltd, UK. All other chemicals not specified were purchased from Sigma (Poole, UK).

### MMTV-NEU-NT tumour model

Female transgenic mice expressing the mutant-activated form of rat NEU (NEU-NT) under transcriptional control of the MMTV promoter (MMTV-NEU-NT mice) were purchased from Charles River, UK. The mating strain is the FVB/N mouse and multiple tumours involving the entire mammary epithelium arise synchronously in the mammary gland area [21] between 18 and 20 weeks of age in 35–50% of the mice.

Mice were maintained under strict inbreeding conditions; health was monitored every 3 months and the presence of the *Neu* transgene was routinely checked by PCR on tail DNA. Animals were anaesthetized with an isoflurane/oxygen mixture. All experiments were performed in accordance with the UK Animals Scientific Procedures Act 1986 and within the NCRI guidelines for the cancer research community concerning the use and welfare of experimental animals in oncology [26]. All possible measures were taken to minimise any pain or discomfort to the animals.

Tumour-bearing mice were anaesthetized, tumour size measured and mice injected intraperitoneally with β-OHB at 500 mg/kg body weight daily for 3 weeks. A control group was injected with PBS daily at 10 ml/kg body weight. Tumour volume was measured weekly. Tumour volume was calculated using the formula *(π/6)(d1 · d2 · d3)* where *d1*, *d2* and *d3* are the three orthogonal diameters measured by callipers to derive an ellipsoidal volume. At day 21, part of the tumour and liver were freeze-clamped for metabolite analysis and western blots and part was placed in 10% neutral buffered formalin (formalin) for histological staining. The liver was used as the normal tissue because the mouse mammary organ is a diffuse, subcutaneous sheet of tissue that cannot be separately freeze-clamped for metabolic studies.

### High-resolution ^1^H and ^31^P MRS of tumour extracts

Part of the freeze-clamped tumour was extracted with four volumes of 6% perchloric acid and centrifuged at 1000 g for 10 mins. The supernatants were neutralised, freeze-dried, reconstituted in deuterium oxide and transferred to 5-mm NMR tubes. MR spectra were acquired at room temperature on a 600-MHz Bruker spectrometer (Bruker Biospin, Coventry, UK).

^*1*^ *H MRS:* The water resonance was suppressed by using gated irradiation centred on the water frequency. TSP (50 μl, 5 mM) was used for chemical shift calibration and quantitation.

^*31*^ *P MRS: *Metal ions were chelated by addition of EDTA (ethylenediaminetetraacetic acid, 50 μl, 60 mM) and MDP (methylenediphosphonic acid, 50 μl, 5 mM) was added to each sample for chemical shift calibration and quantitation.

Metabolite concentrations were determined by peak integration, normalised to the peak integral of the respective internal standard (TSP for ^1^H and MDP for ^31^P MRS).

### Histology

The formalin-fixed part of the tumour was embedded in paraffin wax and 3-μm sections cut de-waxed and rehydrated as standard. Slides were stained immunohistochemically for CD31, Ki-67 and cleaved caspase 3 using a biotin-free bond polymer refine detection kit (Leica Cat No DS9800) and the automated Bond staining platform. Additional slides were stained for TUNEL using a hybrid Bond-manual-Bond protocol.

*CD31 staining:* Antigen retrieval was by proteinase k enzyme digestion (AR 9551, Leica) at 37 °C for 10 mins. The primary antibody was the rat anti-mouse CD31, 1:400 (BD Pharmingen, 553370), and the secondary antibodies were goat anti-rat 1:500 (Jackson Immuno Research 12-005-167) and rabbit anti-goat 1:500 (Jackson Immuno Research 305-005-045).

*Ki-67 staining:* Antigen retrieval was by sodium citrate digestion at 100 °C for 20 min, and the primary antibody was the rabbit anti Ki-67, 1:1000 (Bethyl Laboratories, IHC-00375).

*Cleaved caspase 3 staining:* Antigen retrieval was by Tris EDTA for 20 min at 100 °C; the primary antibody was rabbit mAb 9664 used at 1:200 (Cell Signalling Technology).

*TUNEL staining:* Slides were pre-treated at 37 °C for 10 min in Bond enzyme concentrate containing a proteolytic enzyme and stabiliser (AR9551) followed by a peroxidase block for 5 min (Leica kit). Slides were then processed manually for the hybridisation step in rTdT reaction mix at 1:1000 for 60 min at 37 °C (Promega kit), for 3 × 15 min stringency washes using SSC at 1:20 (Promega kit) and 30-min incubation with SA-HRP at 1:500 (Leica kit). They were returned to the Bond platform for subsequent 10-min refining with 3-3′Diaminobenzidine (DAB) (Leica kit) and 5-min staining with haematoxylin (Leica kit). Two sequential slides were cut from each block for positive and negative staining; for negative staining the enzyme in the reaction mix was replaced by distilled water. Values for the negative slide were subtracted from those for the adjacent positive slide after scanning and analysis.

The DAB enhancer used for all staining was AR 9432 (Leica).

*Analysis:* Slides were scanned with an Aperio Scanscope AT2 at ×20 magnification and resolution of 0.5 microns per pixel. Automated analysis of CD31 (microvessel density) staining was performed using the Aperio Imagescope algorithm “Microvessel Analysis version 1”.

Halo software (Indica Labs) “Cytonuclear version 1.4” was used for automated analysis of Ki-67 staining to identify nuclei, count and grade them according to stain intensity and for automated analysis of CC3 and TUNEL staining using positive pixel count to measure the total stained area. All algorithms excluded necrotic regions and the lacunae typical of these tumours [27, 28]; hence, all values for histological parameters were expressed per viable tissue area.

### Western blotting

Total cell extracts from freeze-clamped, pulverised tissues were obtained by incubation on ice for 10 min in cell lysis buffer (RIPA, Pierce) supplemented with proteinase inhibitors (1 × Complete (Roche), 1:500 PI cocktail (Sigma)) and 10 mM sodium butyrate (Sigma)). Lysates were sonicated (Bioruptor; 3 × 30 s, high setting) and spun at 13000 rpm, 4 °C for 30 s. Thirty microgram protein extracts were run on 4–20% Bis-Tris gradient gels (Invitrogen), transferred onto PVDF membranes (GE Healthcare) and stained with Ponceau S (Sigma). The blotted membranes were cut at ~40 KDa and blocked with 3% skimmed milk in TBS-T (TBS/0.02% Tween-20) for 1 h at room temperature. All antibody incubations were in 1% milk/TBS-T for 1 h at room temperature and washes between antibodies in TBS-T. The bottom half was incubated with rabbit anti-H3Ac (Millipore, 06-599, 1:10,000) and the top half with rabbit anti-LaminB1 (Abcam, ab16408, 1:1000). After 3 × 5 min washes, top and bottom half membranes were incubated with donkey anti-rabbit IR-Dye 800 (LI-COR, 926-32213), washed and scanned on a LI-COR Odyssey scanner. Fluorescence intensity signals were quantified with ImageQuant (GE Healthcare).

### Cell culture and ^1^H NMR analysis

A cell line of C-*neu*/HER2-induced mammary carcinoma cells was established in-house from a tumour mass excised from an MMTV-NEU-NT mouse. Tumour cells were cultured in high glucose Dulbecco’s modified eagle’s medium (DMEM) (4.5 g/L, Gibco 41966) supplemented with 10% foetal calf serum (FCS), 1000 U/ml penicillin and 100 μg/ml streptomycin. Before treatment with β-OHB, cells were seeded in 6-well plates at a density of 7 × 10^5^ per well containing low-glucose DMEM (1 g/L, Gibco 12320) supplemented with 10% FCS, 1000 U/ml penicillin and 100 μg/ml streptomycin. β-OHB was added at concentrations 0, 0.5, 5 and 10 mM. Twenty-four hours after treatment, the medium in each well was analysed for glucose and lactate by ^1^H MRS on a 600-MHz Bruker Spectrometer with DSS (4, 4-dimethyl-4-silapentane-1-sulfonic acid) (10 μl, 120 mM) as a standard for chemical shift calibration and quantitation. Cells in each well were removed with 0.05% trypsin and counted on a Luna^TM^ automated cell counter using trypan blue exclusion for viability. Results are expressed as micromole glucose consumed or micromole lactate produced/10^6^ viable cells/24 h.

### Statistical analysis

The solid tumour, tumour extract, tumour cell and histological data are presented as the mean ± SEM. Two-tailed unpaired Student’s *t* tests were used for comparison of tumour volumes, metabolite concentrations and histological data, and a *p* value of <0.05 was considered to be statistically significant.

## Results

### Effect of β-OHB on tumour volume and growth rate

**Fig. 1:**
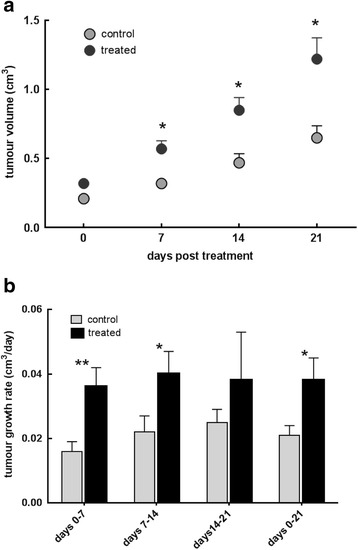
**a** Tumour volume in MMTV-NEU-NT tumour-bearing control mice and mice treated with β-OHB (500 mg/kg), injected ip daily for 3 weeks. Control: *N* = 25 tumours (18 mice), Treated: *N* = 23 tumours (19 mice); * *p* < 0.005. **b** Tumour growth rates (cm^3^/day) post-treatment with β-OHB (500 mg/kg), injected ip daily for 3 weeks during week 1 (days 0–7), week 2 (days 7–14), week 3 (days 14–21) and overall, weeks 1–3 (days 0–21); ** *p* < 0.005, * *p* < 0.05

The MMTV-NEU-NT mouse strain develops multiple tumours within the mammary gland between 18 and 20 weeks of age. The mice that were treated with ip β-OHB showed a significant increase in tumour growth rate (Fig. 1a) over the 3-week treatment (*p* = 0.04) with significant increases during week 1 and week 2 (*p* = 0.003 and *p* = 0.035 respectively) (Fig. 1b). The tumour growth rate in the control mice over the 3-week period was 21 ± 3 mm^3^/day compared to 38 ± 7 mm^3^/day in the treated cohort (*p* = 0.04, Fig. 1b). The mean doubling time decreased from 14.2 ± 5 days in the control group to 11.6 ± 4.7 days in the treated cohort (*p* = 0.11, Fig. 1a). Tumour volume increased over the 3-week period from 0.21 ± 0.02 to 0.65 ± 0.09 cm^3^ in the control and 0.32 ± 0.03 to 1.22 ± 0.15 cm^3^ in the treated cohorts (Fig. 1a).

### Effect of β-OHB on tumour branched chain amino acids, glutamine and glycine

**Fig. 2:**
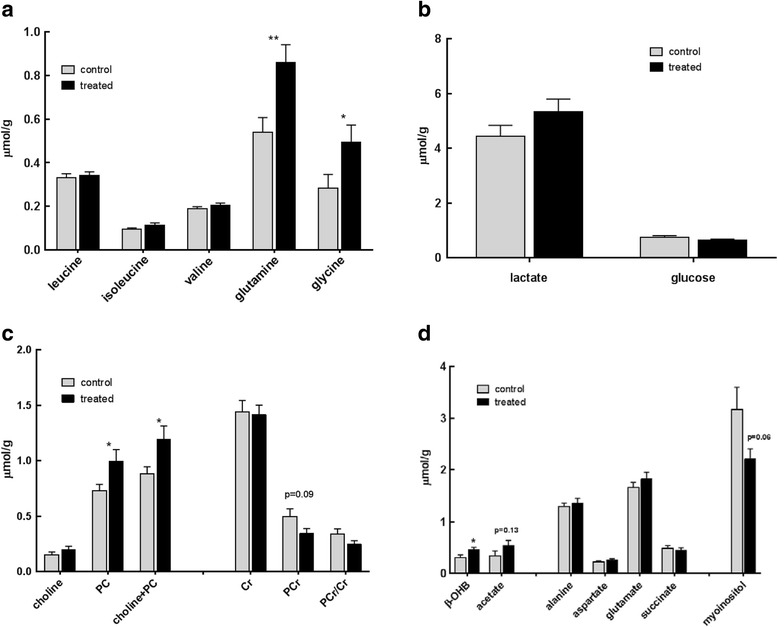
Metabolites (μmol/g) measured by ^1^H MRS in tumour extracts at day 21 from MMTV-NEU-NT tumour-bearing control mice and mice treated with β-OHB (500 mg/kg), injected ip daily for 3 weeks. **a** Branched chain amino acids, glutamine and glycine,** *p* < 0.005, * *p* < 0.05. **b** Lactate and glucose. **c** Choline-containing metabolites: choline, phosphocholine (PC), total of choline + phosphocholine; creatine containing metabolites: creatine (Cr), phosphocreatine (PCr), ratio of PCr/Cr. * *p* < 0.05 (D) β-OHB and acetate; amino acids alanine, aspartate and glutamate; succinic acid and myo-inositol. Control: *N* = 22 tumours (13 mice); Treated: *N* = 33 tumours (19 mice), *p < 0.05. **d** other metabolites

**Fig. 3:**
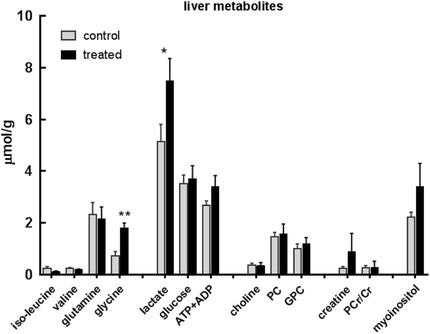
Metabolites (μmol/g) measured by ^1^H MRS in liver extracts at day 21 from MMTV-NEU-NT tumour-bearing control mice and mice treated with β-OHB (500 mg/kg) injected ip daily for 3 weeks. Isoleucine, valine, glutamine, glycine, lactate, glucose, ATP + ADP, choline, phosphocholine (PC), glycerophosphocholine (GPC), creatine (Cr), phosphocreatine (PCr), myo-inositol. Control: *N* = 12; Treated: *N* = 8; *p < 0.05, ** *p* < 0.01

The tumours of mice treated with β-OHB contained significantly higher concentrations of glutamine and glycine but no change in the branched chain amino acids (Fig. 2a). The glutamine levels increased from 0.54 ± 0.07 in the controls to 0.86 ± 0.08 μmol/g in the treated tumours (*p* = 0.004) and the glycine from 0.28 ± 0.06 to 0.49 ± 0.08 μmol/g (*p* = 0.04, Fig. 2a). The increases in the branched chain amino acids (leucine, isoleucine and valine) were not significant (*p* > 0.14). The livers of the treated mice showed a significant increase in glycine from 0.73 ± 0.16 in the controls to 1.81 ± 0.19 μmol/g (*p* = 0.008) after β-OHB treatment, whereas glutamine or branched chain amino acids did not show a significant change (Fig. 3).

### Effect of β-OHB on tumour glycolytic intermediates, choline, creatine and phosphorus-containing metabolites

**Fig. 4:**
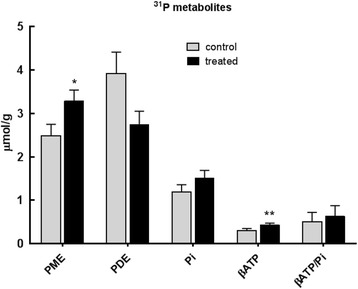
Phosphorus containing metabolites (μmol/g), measured by ^31^P MRS, in tumour extracts at day 21 from MMTV-NEU-NT tumour-bearing control mice and mice treated with β-OHB (500 mg/kg), injected ip daily for 3 weeks. Phosphomonoesters (PME = phosphocholine (PC) + phosphoethanolamine (PE)), phosphodiesters (PDE = glycerophosphocholine + glycerophosphoethanolamine), inorganic phosphate (Pi), and β-ATP/Pi. Control: *N* = 8 to 13 (6 mice); Treated: *N* = 13–18 (12 mice), *p ≤ 0.05

On treatment with β-OHB, the tumour lactate increase from 4.44 ± 0.39 to 5.33 ± 0.47 μmol/g and glucose decrease from 0.73 ± 0.07 to 0.62 ± 0.05 μmol/g were not significant (*p* = 0.15 and 0.23 respectively) (Fig. 2b). However, there was a significant increase in liver lactate from 5.13 ± 0.68 to 7.49 ± 0.86 μmol/g (*p* = 0.032) (Fig. 3). There was also a significant increase in tumour tCho (*p* = 0.03) caused chiefly by an increase in PC from 0.73 ± 0.06 to 1.0 ± 0.11 μmol/g (*p* = 0.03). The decrease in PCr from 0.5 ± 0.07 to 0.34 ± 0.04 μmol/g was not significant (*p* = 0.09) (Fig. 2c). ^31^P MRS also showed a significant increase in the PME peak, consisting mainly of PE and PC, from 2.48 ± 0.27 μmol/g in the control to 3.28 ± 0.25/g in the treated tumours (*p* = 0.04). There was a small but significant increase in the ATP levels, from 0.3 ± 0.05 to 0.43 ± 0.04 μmol/g (*p* = 0.05) (Fig. 4).

### Effect of β-OHB on tumour ketone body levels, myo-inositol and amino acids alanine, aspartate and glutamate

Levels of β-OHB and acetate were higher in the tumours of treated mice compared to controls (*p* = 0.04 and *p* = 0.13 respectively) whereas myo-inositol was lower in the treated tumours (*p* = 0.06) (Fig. 2d).

### Effect of β-OHB on global histone H3 acetylation levels of MMTV-NEU-NT tumours and livers

**Fig. 5:**
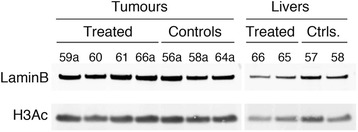
Western blots for global histone H3 acetylation (H3Ac) and LaminB in MMTV-NEU-NT tumour and liver extracts. Tissues from treated mice (daily ip injection of 500 mg/kg β-OHB) and control mice were freeze-clamped at day 21. A representative blot is shown. Across the entire sample set, the mean (±se) intensity of global H3Ac relative to LaminB in treated tumours (*n* = 10) was 0.713 (±0.005) compared to 0.758 (±0.034) in the control tumours (*n* = 15), *p* = 0.22. In the treated livers, the mean intensity was 0.706 (*n* = 2) compared to 0.711 ± 0.002 (*n* = 4) in the control livers

We looked at the effect of β-OHB on global histone H3 acetylation by western blotting and found that the tumours and livers from mice treated with β-OHB showed no significant change in the pan-histone 3 acetylation levels (H3ac) compared to control tumours and liver respectively (Fig. 5). Lamin B was used as the loading control. The relative intensity of H3ac in the treated tumours was 0.713 ± 0.005 (*n* = 10) compared to 0.758 ± 0.034 (*n* = 15) in the control tumours (*p* = 0.22). In the treated and control livers, the mean intensity was 0.71 in each case.

### Effect of β-OHB on tumour vasculature, proliferation and apoptosis markers as measured by immunohistochemistry

**Fig. 6:**
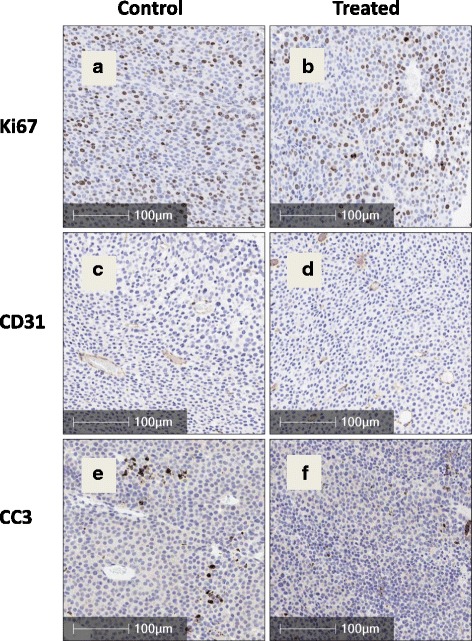
Ki-67 (proliferation marker, panels **a** and **b**), CD31 (measure of vascularity, panels **c** and **d**) and cleaved caspase 3 (apoptosis marker, panels **e** and **f**) immunohistochemical staining of tumour sections at day 21 from control mice (*left*) and mice treated with β-OHB (500 mg/kg), injected ip daily for 3 weeks (*right*) (*Bars* show 100 μm). See Table 1 for quantitative analysis

There was no significant change in tumour vasculature as measured by CD31 immunohistochemistry (97.2 ± 12.2 in the control compared to 133.2 ± 18.0 vessels/mm^2^ in the treated tumours, *p* = 0.13) or in tumour proliferation as measured by Ki-67 staining (28.9 ± 4.0% positive in the control compared to 21.7 ± 3.9% positive in the treated, *p* = 0.22). Similarly, staining for cleaved caspase 3 and TUNEL showed no significant differences between treated and control tumours (Table 1). Fig. 6 shows representative images of markers for vasculature, proliferation and an apoptosis marker.

**Table 1:** Quantitative histological analysis of viable areas of tumour sections (control and β-OHB treated) stained for CD31 (microvessel density as number of vessels/mm^2^), Ki-67 (proliferative index as % positive cells), cleaved caspase 3 and TUNEL (percent positively stained area)—see Fig. 6. Values are mean ± SEM

	CD31 (microvessel density: vessels/mm^2^)	Ki-67 Index (% positive cells)	CC3 (% positively stained area)	TUNEL (% positively stained area)
Control (*N* = 7)	97.2 ± 12.2	28.9 ± 4.0	1.54 ± 0.36	3.30 ± 1.08
β-OHB treated (*N* = 8)	133.2 ± 18.0	21.7 ± 3.9	0.83 ± 0.26	2.55 ± 1.40
*p* (*t* test)	0.13	0.22	0.13	0.69

### Effect of β-OHB concentration on β-OHB uptake, glucose uptake and lactate output in MMTV-NEU-NT tumour cells in culture

C-*neu*/HER2 cells were cultured in the presence of 0, 0.5, 5 and 10 mM β-OHB and the growth medium was analysed for β-OHB, glucose and lactate at 0 and 24 h. The cells secreted about 33% more lactate than they consumed glucose. Allowing for the fact that two molecules of lactate are produced from each glucose, this implies that about 2/3 of the glucose was metabolised to lactate via glycolysis. When the cells were cultured in medium containing 0.5 mM β-OHB, a level similar to that measured in treated tumours in vivo, the uptake of β-OHB was 260 ± 71 nmol/10^6^ cells/24 h. When the cells were cultured in medium containing 5 mM β-OHB, the β-OHB uptake was 420 ± 264 nmol/10^6^ cells/24 h and at 10 mM β-OHB, it was 834 ± 551 nmol/10^6^ cells/24 h. However, these higher β-OHB uptake values at higher β-OHB concentrations were not significantly different from those at 0.5 mM β-OHB because of the wide scatter in the results.

**Fig. 7:**
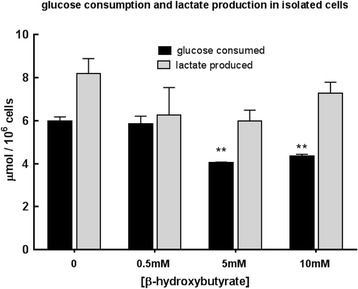
Effect of β-OHB (0, 0.5, 5, 10 mM) on glucose consumption and lactate production (μmol/10^6^ cells/24 h) at 24 h when MMTV-NEU-NT cells were incubated in low-glucose (1 g/L) DMEM. *N* = 3, **p < 0.005

When the cells were cultured at 0.5 mM β-OHB, there was no significant difference in glucose consumption compared with culture in medium without β-OHB. However, at the higher β-OHB levels of 5 mM and 10 mM, a significant decrease in glucose consumption was observed relative to controls (*p* < 0.005) (Fig. 7). Surprisingly, in view of the decreased glucose consumption by cells grown in 5 or 10 mM β-OHB, there was no significant difference in lactate production in any of the treated cells when compared to controls (Fig. 7).

## Discussion

The results presented here indicate that daily ip injections of the ketone body β-OHB over a 3-week period significantly increased tumour growth rate in this autochthonous mouse mammary tumour model, although no changes were seen in proliferative fraction, blood vessel density or apoptosis. This suggests that the cycle time of the tumour cells was decreased by the treatment. Calculation of the cell cycle time for each group from the respective doubling time (Fig. 1a) and proliferative index (Table 1) gives a cycle time of 4.7 days for the controls and 3.6 days for the treated tumours.

The growth-promoting properties of β-OHB seen in this study are similar to those observed by Lisanti’s group [8–10] in breast cancer xenografts and cultured breast cancer cells, but contrary to the growth-inhibitory effects of KB that have been reported in other cancers and in metastasis [3, 5, 7]. Like those of Lisanti’s group, our data were obtained from a breast cancer model, which suggests that the opposing responses of tumours to ketones may be tumour-type dependent, rather than a general phenomenon, and may perhaps be dictated by the different genetic drivers of tumours arising in different tissues.

Since β-OHB is closely related to butyrate, our results in the mammary tumours could be interpreted by a mechanism analogous to the “butyrate paradox” described by Donohoe et al. [16] that explains the opposing effects of butyrate on inhibiting growth of cancer cells and promoting growth of normal cells. They argue that cancer cells that show the Warburg effect favour glycolysis over oxidative metabolism of substrates such as butyrate; butyrate therefore accumulates and inhibits cancer cell proliferation by acting as an HDAC inhibitor. In contrast, butyrate oxidation by normal cells reduces butyrate concentrations below the level needed for HDAC inhibition; KB oxidation will then be an energy source for non-cancerous cells, and can allow them to grow faster. It seems likely that in cancerous cells that do not display the Warburg effect (i.e., those that can oxidise glucose and other substrates and do not rely exclusively on glycolysis for ATP phosphorylation) β-OHB could be metabolised in the same way as in the normal cells studied by Donohoe et al., so that its concentration will fall below that required for growth-inhibitory HDAC inhibition and the ATP generated will facilitate proliferation.

Our treatment regime resulted in β-OHB levels of 0.46 ± 0.05 μmol/g within the tumours (Fig. 2d), a concentration that had no effect on histone acetylation (Fig. 5). Was the low β-OHB concentration due to it being metabolised? Our results on cultured C-neu/HER2 cells incubated with 0.5 mM β-OHB (approximately the concentration that we had found in solid tumours) showed that the cells took up 0.26 ± 0.71 μmol β-OHB/10^6^ cells/24 h, implying that metabolism of β-OHB would indeed have reduced its concentration in the tumours (Table 2).

**Table 2:** Effect of β-OHB in the culture medium on uptake of β-OHB, uptake of glucose and output of lactate. Uptake and output data are the means from the experiment shown in Fig. 7, *N* = 3

β-OHB in medium (mM)	β-OHB uptake^a^	Glucose uptake^a^	Lactate output^a^	β-OHB ATP as % of lactate ATP
0	–	6.0	8.2	–
0.5	0.26	5.8	6.3	54%
5	0.42	4.0	6.0	91%
10	0.83	4.3	7.3	148%

^a^μmol/10^6^ cells/24 h

In the absence of β-OHB the output of lactate from the C-neu/HER2 cells (8.2 μmol/10^6^ cells/24 h (Table 2)) was equivalent (since two lactates are formed from each glucose) to about 70% of their glucose uptake; thus, around 30% of the glucose would have been available for anabolic metabolism or, if the cells were not fully glycolytic, for oxidative respiration. Addition of 0.5, 5.0 or 10.0 mM β-OHB to the culture medium had little effect on lactate output (Table 2) so it seems likely that the ATP production from β-OHB oxidation was additional to that obtained from glycolysis. Using Murray’s estimate of 13 ATP per β-OHB [29], an uptake of 0.26 μmol β-OHB/10^6^ cells/24 h (as observed when 0.5 mM β-OHB was added to the culture medium) would phosphorylate 3.38 μmol ATP compared to the 6.3 μmol ATP obtained by glycolytic formation of 6.3 μmol lactate/10^6^ cells/24 h. Thus, if the cultured cancer cells had been relying entirely on glycolytic ATP phosphorylation (i.e., the Warburg effect) in the absence of β-OHB, then provision of 0.5 mM β-OHB (approximately the concentration found in the solid tumours when the mice were injected with β-OHB) would have increased their ATP output by about 50% (see Table 2).

When the cells were incubated with 5 mM β-OHB (i.e., a 10-fold higher concentration than was observed in the solid tumours) β-OHB uptake increased to 0.42 μmol/10^6^ cells/24 h whereas glucose consumption decreased to 4.0 μmol/10^6^ cells/24 h and lactate output remained at 6.0 μmol/10^6^ cells/24 h, indicating that 75% of the glucose uptake was used in glycolysis (Fig. 7) and that the ATP output from β-OHB oxidation would have been around 90% of that provided by glycolysis. The most extreme case we considered was culture in 10 mM β-OHB (about 20-fold higher than the concentration observed in the solid tumours) when the β-OHB uptake increased to 0.83 μmol β-OHB/10^6^ cells/24 h and lactate output to 7.3 μmol/10^6^ cells/24 h but the glucose consumption stayed at 4.3 μmol/10^6^ cells/24 h (Fig. 7), implying that around 85% of the glucose uptake was consumed in glycolysis and that β-OHB oxidation would have provided about 50% more ATP than glycolysis (Table 2).

Taken all in all, it is evident from the cell culture studies that the C-neu/HER2 cancer cells would have substantially increased their ATP output by oxidising β-OHB, even when it was present at the low concentration observed in the solid tumours in vivo, and that oxidation of β-OHB seems to have substituted for oxidative metabolism of glucose. If that effect also took place in the solid tumours (i.e., if they were able to increase their ATP output substantially by oxidising β-OHB, and perhaps also redirect the glucose from oxidation into anabolic synthesis, both of which effects would be growth-promoting), then it seems likely that these mechanisms would have reduced the concentration of β-OHB to a level that did not inhibit HDAC and that the cells would have had more ATP and perhaps also glycolytic intermediates for faster growth. The enhanced growth rate and absence of histone acetylation change would thus constitute a β-OHB paradox.

In addition, Donohoe et al. [16] showed that butyrate (0.5 mM) inhibited ATP citrate lyase, which also induced a stimulatory effect on cell growth, though reduced by a factor of 3, which they attributed to butyrate having a stimulatory effect by increasing acetyl-CoA production for lipid biosynthesis and/or acetylation of lysine residues. They also showed that there was no increase in histone H3 acetylation with low doses of butyrate when the Warburg effect was blocked. Their results demonstrate elegantly that the Warburg effect can be a cause rather than an effect of changes in histone acetylation and that cancer metabolism can drive the epigenetic profile of cancer cells away from the cell of origin and contribute to tumourigenesis. Another recent study demonstrates that the rate of glycolysis quantitatively mediates specific histone acetylation sites, and the authors suggest that a possible function of the Warburg effect is to confer specific signalling effects on cells by altering the levels of multiple key metabolites that serve as cofactors and substrates for reactions involved in post-translational protein modifications [30]. We did not consider these latter issues in our study.

The tumours in the β-OHB-treated mice showed a number of other features characteristic of enhanced growth rate. There were significant increases in the ^1^H MRS peaks for choline and PC plus choline, and also in the ^31^P MRS PME peak, which includes PC, while the PDE peak, which includes GPC, was lower in the treated tumours but the change was just outside the significant range (*p* = 0.06). These effects are all in keeping with the upregulated choline metabolism that is characteristic of progressing tumours [31, 32]. PC is both a precursor and a breakdown product of phosphatidylcholine, the main phospholipid component of cell membranes, while GPC is a breakdown product. During the increased rates of membrane turnover that are characteristic of tumour growth, all these signals tend to be elevated, both in the tumour cells and host cells in the tumour microenvironment, a phenomenon that is termed the choline phenotype. Elevation of tCho levels has been associated with aggressiveness in breast cancer [32] and prostate cancer [33], and it has been used in the grading of brain tumours [34]. In breast and ovarian cancers, the low PC and high GPC in non-malignant cells change to high PC and low GPC after malignant transformation [31, 35]. Our results are therefore consistent with induction of the choline phenotype in the treated tumours and thus with their increased growth rate.

Treatment of the tumours with β-OHB also resulted in significantly increased concentrations of the amino acids glutamine and glycine (Fig. 2a). The carbon skeletons of glycine (via conversion to serine and then pyruvate) and glutamine (via conversion to glutamate and then α-ketoglutarate, passage through the tricarboxylic acid (TCA) cycle and then formation of pyruvate) can be ultimately converted to acetyl-CoA and oxidised in the tricarboxylic acid cycle. This is the same pathway as is utilised for β-OHB oxidation, so perhaps the elevated concentrations of glycine and glutamine in β-OHB-treated animals can be explained by competition for that breakdown pathway. Glutamine, a versatile nutrient that has a role both in oxidative phosphorylation and macromolecular synthesis, is required for survival and growth of a variety of tumours [36]. It also modulates proliferative signalling mechanisms such as the activation of the serine/threonine kinase mTOR [37]. Not all cancer cells need an exogenous supply of glutamine, however. Breast cancer cells have shown systematic differences in glutamine dependence, with basal-type cells being glutamine dependent and luminal-type cells being glutamine independent [38]. An analysis of glutamine metabolism in lung and liver tumours revealed that both tissue of origin and the cancer-inducing oncogene influence whether tumours produce or consume glutamine [39]. Although tumours differ in their need for glutamine, the increase in glutamine concentration that we observed after β-OHB treatment is consistent with increased energy requirements and macromolecular synthesis in progressing tumours.

The recent surge of interest in the study of metabolic processes in cancer has focussed mainly on glucose and glutamine metabolism, but the serine and glycine pathways are now also thought to be perturbed in tumours [40]. In a recent metabolic flux analysis across a panel of CL-60 tumour lines, Jain et al. showed an unexpectedly increased reliance on glycine metabolism in proliferating cells, a phenotype that was not observed in proliferating non-transformed cells [41]. They showed that glycine is utilised for de novo purine nucleotide biosynthesis while others have shown that glycine and related metabolites including serine and threonine are central to cellular transformation [42, 43]. The significant increase in glycine we observed in tumours treated with βOHB is thus in keeping with the phenotype of proliferating and/or transformed tissue. The decrease in myo-inositol with increase in tumour growth rate that we observed is also in keeping with what has been previously observed in breast cancer where myo-inositol is lower in breast tumour tissue compared to normal tissue [44]. The role of myo-inositol in cancer is unclear but it is thought to be involved in hormone signal transduction and as an osmoregulator at different stages of malignant transformation.

We also performed metabolic analyses on the liver, to see whether β-OHB administration affected a normal tissue (the mouse mammary organ cannot be freeze-clamped, so we used the liver as our control). In the β-OHB-treated animals we observed significant increases in liver lactate and glycine. A possible explanation for the absence of a rise in liver glucose despite the increase in lactate is that glucose in the liver is thought to be extracellular, because it is immediately phosphorylated when taken up into liver cells and is exported from them as soon as it is dephosphorylated. Since the mice had been normally fed prior to anaesthesia, it is likely that they were still in a post-absorptive state, so the extracellular glucose concentration would have depended on its rate of uptake from the hepatic portal vein and its rate of output into the liver cells. The liver also takes up lactate for conversion to glucose via gluconeogenesis, and the measured concentration includes both the intracellular and extracellular pools. Thus, since their metabolic pathways are not directly connected, it is not necessarily surprising that we observed an elevated liver lactate in the absence of an elevated liver glucose.

## Conclusions

To the best of our knowledge, this study is the first to test the effect of β-OHB on the growth of a genetically engineered autochthonous tumour model. We found that β-OHB administration accelerated the rate of tumour growth rather than inhibiting it, and that the tumours did not show evidence of enhanced histone H3 acetylation. The metabolic profile of these tumours post-treatment fits with the general phenotype of proliferating tissues, with increase in ATP, glutamine, serine and choline-related metabolites.

We have thus found evidence that spontaneous mouse mammary tumours show a β-hydroxybutyrate paradox in which tumour growth is promoted by β-OHB, probably (a) because oxidative metabolism of β-OHB reduces its concentration below the level at which it could induce inhibition of tumour growth via inhibition of histone deacetylation, (b) because the oxidised β-OHB contributes significantly to the tumour cell’s ATP balance and perhaps also (c) because the glucose spared from oxidation is available for increased anabolic synthesis. This β-hydroxybutyrate paradox could explain the contradictory results in the literature concerning the anti-cancer effects of β-OHB: perhaps the studies that showed an anti-cancer effect of β-OHB [3, 5, 7] used tumour models that preferentially utilised glucose rather than oxidising β-OHB, and thus accumulated high β-OHB concentrations that inhibited HDAC, whereas those that failed to show an anti-cancer effect [8–10] were conducted on tumour models that preferentially oxidised β-OHB, thus enhancing their growth rate and reducing their β-OHB concentration below the level at which it would slow tumour growth by inhibiting histone deacylation.

## Abbreviations

ATP: Adenosine triphosphate
Cr: Creatine
DAB: 3-3′Diaminobenzidine
DMEM: Dulbecco’s modified eagle’s medium
DSS: 4, 4-Dimethyl-4-silapentane-1-sulfonic acid
EDTA: Ethylenediaminetetraacetic acid
FCS: Foetal calf serum
FDG-PET: Fluorodeoxyglucose positron emission tomography
GPC: Glycerophosphocholine
GPE: Glycerophosphoethanolamine
H3ac: Global histone 3-acetylation
HATs: Histone acetyl transferases
HDACs: Histone deacetylases
ip: Intraperitoneally
KB: Ketone bodies
MDP: Methylene diphosphonic acid
MRS: Magnetic Resonance Spectroscopy
NTP: Nucleoside triphosphates
PBS: Phosphate buffered saline
PC: Phosphocholine
PCA: Perchloric acid
PCR: Polymerase chain reaction
PCr: Phosphocreatine
PDEs: Phosphodiesters
PE: Phosphoethanolamine
P_i_: Inorganic phosphate
PMEs: Phosphomonoesters
TBS-T: Tris-buffered saline with Tween-20
TCA: Tricarboxylic acid
tCho: Total choline
TSP: Trimethylsilyl propionate
β-OHB: Beta-hydroxybutyrate
